# Randomized comparative study of 1-Hz transcranial magnetic stimulation (TMS), continuous theta-burst stimulation (cTBS) and sham-TMS for treatment-refractory auditory hallucinations (AH) in schizophrenia

**DOI:** 10.1192/j.eurpsy.2022.1903

**Published:** 2022-09-01

**Authors:** I. Potapov, N. Maslenikov, E. Tsukarzi, S. Mosolov

**Affiliations:** Moscow Research Institute of Psychiatry, Department For Treatment Of Mental Disorders, Moscow, Russian Federation

**Keywords:** schizophrénia, cTBS, auditory hallucinations, TMS

## Abstract

**Introduction:**

Insufficient efficacy of conventional treatment of auditory hallucinations (AH) in schizophrenia supports rising interest to brain stimulation techniques including transcranial magnetic stimulation (TMS). Left temporo-parietal cortex (TP3) is involved in emergence of AH, thus neuromodulation of this area might be reasonable.

**Objectives:**

Comparison of efficacy and tolerability of 2 protocols of TMS (1 Hz and cTBS) over TP3 and sham-TMS for treatment resistant AH in schizophrenia.

**Methods:**

76 schizophrenia (ICD-10 - F20) patients with prominent AH (PANSS P3 ≥ 4, AHRS ≥ 15), who had failed to respond to previous antipsychotic treatment, were randomized into 3 groups: 1) 1 Hz TMS (30 patients); 2) cTBS (25 patients); 3) Sham-TMS (21 patients). Sessions were performed 5 days a week for 3 weeks. Antipsychotic medication was continued throughout the study. Patients were assessed weekly with PANSS, AHRS, CDSS, CGI-S by blinded raters. The criterion of efficacy was 30% AHRS score reduction after 3 weeks of treatment.

**Results:**

The number of responders were 13 (43,3%) in 1 Hz TMS group, 14 (56%) – in cTBS group, 4 (19,1%) in sham-TMS group. There was no statistically significant difference in efficacy between 1 Hz TMS and cTBS, but each of the active protocols was more effective than sham-TMS. Treatment was generally well tolerated in all groups, nobody was discontinued the study due to adverse events.

**Conclusions:**

Both protocols of TMS (1 Hz and cTBS) over TP3 are safe and effective in the treatment of schizophrenic patients with pharmacotherapy resistant AH. Further studies are needed.

**Disclosure:**

No significant relationships.

